# Preparation and Characterization of Nanofibrous Membranes Electro-Spun from Blended Poly(l-lactide-co-ε-caprolactone) and Recombinant Spider Silk Protein as Potential Skin Regeneration Scaffold

**DOI:** 10.3390/ijms232214055

**Published:** 2022-11-14

**Authors:** Suyang Wang, Hongnian Zhu, Qing Meng

**Affiliations:** College of Biological Science and Medical Engineering, Donghua University, Shanghai 201620, China

**Keywords:** recombinant spider silk protein, electrospinning, skin regeneration, nano scaffolds

## Abstract

Biomaterial scaffolding serves as an important strategy in skin tissue engineering. In this research, recombinant spider silk protein (RSSP) and poly(L-lactide-co-ε-caprolactone) (PLCL) were blended in different ratios to fabricate nanofibrous membranes as potential skin regeneration scaffolds with an electro-spinning process. Scanning electron microscopy (SEM), water contact angles measurement, Fourier transform infrared (FTIR) spectroscopy, wide angle X-ray diffraction (WAXD), tensile mechanical tests and thermo-gravimetric analysis (TGA) were carried out to characterize the nanofibrous membranes. The results showed that the blending of RSSP greatly decreased the nanofibers’ average diameter, enhanced the hydrophilicity, changed the microstructure and thermal properties, and could enable tailored mechanical properties of the nanofibrous membranes. Among the blended membranes, the PLCL/RSSP (75/25) membrane was chosen for further investigation on biocompatibility. The results of hemolysis assays and for proliferation of human foreskin fibroblast cells (hFFCs) confirmed the membranes potential use as skin-regeneration scaffolds. Subsequent culture of mouse embryonic fibroblast cells (NIH-3T3) demonstrated the feasibility of the blended membranes as a human epidermal growth factor (hEGF) delivery matrix. The PLCL/RSSP (75/25) membrane possessed good properties comparable to those of human skin with high biocompatibility and the ability of hEGF delivery. Further studies can be carried out on such membranes with chemical or genetic modifications to make better scaffolds for skin regeneration.

## 1. Introduction

Skin primarily serves as a protective barrier against the environment [1,2,3], having a physical structure that renews itself every 2 to 3 weeks [1]. Skin regeneration is important for massive skin loss cases [4] which may result in significant disability or even death [2,5,6,7]. The reconstruction of a complete tissue-engineered skin featuring both the epidermis and the dermis is the ultimate goal to improve healing quality and avoid scar formation [8]. In vitro, fibroblasts cannot be cultured on a plastic dish in conventional culture conditions as keratinocytes. Most methods developed to reconstruct a dermis rely on the culture of fibroblasts in a scaffold to build a three-dimensional tissue [8]. For many skin regeneration scenes, biomaterial is the key component [2,9].

Some chemical polymers have been used to fabricate scaffolds for skin-tissue engineering. One of them is poly(l-lactide-co-ε-caprolactone) (PLCL) [10,11,12] which possesses nontoxic, tissue-compatible and biocompatible properties [13,14,15,16] and has been proved to be a benign scaffold material for other tissue engineering [13,17,18,19]. Existing treatments mostly prefer natural-origin biomaterials over synthetic materials because the former have better biocompatibility [8]; they can mimic some features of the extracellular matrix and thus have the potential to direct the migration, growth and organization of cells during tissue regeneration and wound healing [2]. Collagen [2,20], gelatin [21,22], fibrin [23] and chitosan [21,24] have been used to prepare skin regeneration matrix with different approaches[25]. One of them is to fabricate electro-spun membranes which possess the ability to offer topographic guidance [26]; their spatial structures have effects on cell proliferation and differentiation in 3D directions [27]. However, natural-origin materials present more or less serious drawbacks such as inadequate mechanical properties, limited processability [2] and safety risks. For example, studies show that the biological effects of collagen-based matrices are compromised by its poor mechanical properties leading to faster contraction of the grafts [28,29]. In addition, the collagen- and fibrin-based matrices are costly, creating a huge gap between need and availability [30,31]. Collagen and gelatin are mainly isolated from animal tissues, which may be unsafe because of the potential for viral and prior contamination [32]. New biomaterials for skin regeneration remain to be explored.

Natural spider silk, a biomaterial since ancient times [33], known for its excellent mechanical properties [34], biocompatible and biodegradable functions [35], has been proved capable of enhancing skin regeneration [5,36]. However, it is not practical to use natural spider silk for wide-scale applications because natural spider silk cannot be harvested in large amounts due to the cannibalistic, territorial habit of most spiders and the extremely low silk production capabilities [37]. Synthetic spider silk is an ideal substitute for natural spider silk, however. Nowadays, recombinant spider silk protein can be produced in a variety of heterogenous host organisms [38,39,40,41,42]; it can be harvested in large quantities by using low-cost genetically-altered bacteria [43]. Moreover, recombinant spider silk protein can be assembled into a variety of morphologies [44] with a great range of properties [45]. It can also be chemically [46] or genetically modified [44,47]. To our knowledge, there have been few reports on electro-spun recombinant spider silk protein scaffold for skin regeneration[48,49,50,51].

In this paper, recombinant spider silk protein [52] (RSSP, MW: 93.4 kDa) was blended with PLCL (poly(l-lactide-co-ε-caprolactone)) in different ratios to fabricate PLCL/RSSP nanofibrous membranes using the electro-spinning method. Scanning electron microscopy (SEM), water contact angles measurement, Fourier transform infrared (FTIR) spectroscopy, wide angle X-ray diffraction (WAXD), tensile mechanical tests and thermo-gravimetric analysis (TGA) were used to characterize the PLCL/RSSP nanofibrous membranes. Hemolysis assays, cytocompatibility assays and degradation testing were carried out on a nanofibrous membrane to confirm its potential as skin regeneration scaffold. We also investigated the application of our nanofibrous membrane as a human epidermal growth factor (hEGF) loading matrix. The results gave some theoretical information for further studies to make better scaffolds for skin-tissue regeneration.

## 2. Results and Discussions

### 2.1. Fabrication and Morphology of the PLCL/RSSP Nanofibrous Membranes

**Figure 1:**
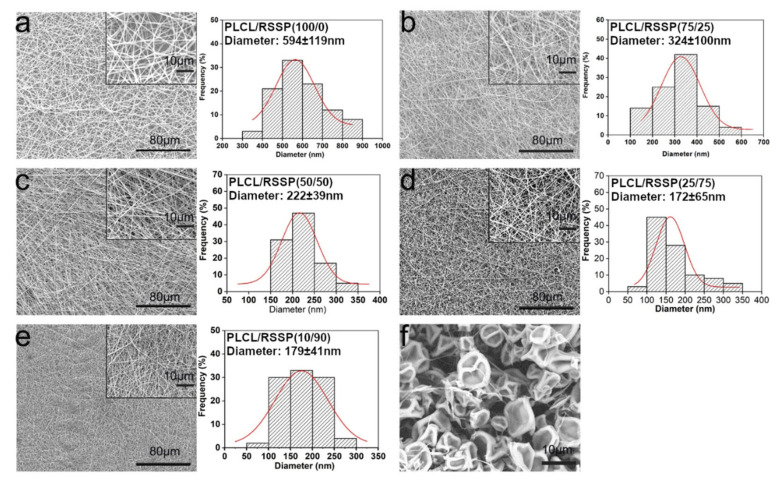
SEM images and diameter distribution histograms of nanofibrous membranes. (**a**) PLCL/RSSP (100/0), (**b**) PLCL/RSSP (75/25), (**c**) PLCL/RSSP (50/50), (**d**) PLCL/RSSP (25/75), (**e**) PLCL/RSSP (10/90) (**f**) PLCL/RSSP (0/100).

Nanofibrous membranes were fabricated from the blended PLCL and RSSP solutions with PLCL/RSSP ratios of 100/0, 75/25, 50/50, 25/75 and 10/90 (*w*/*w*). They were named as PLCL/RSSP (100/0), PLCL/RSSP (75/25), PLCL/RSSP (50/50), PLCL/RSSP (25/75) and PLCL/RSSP (10/90). The nanofibrous membranes showed a three-dimensional structure of randomly-oriented nanofibers (Figure 1a–e). The surface morphology and the diameter distributions of the nanofibers were observed by SEM (Figure 1a–e). In this study, 100% RSSP could not be spun into nanofibrous membrane (Figure 1f).

The surface of the nanofibers was smooth, and the diameters of these nanofibers maintained a relatively narrow distribution range (Figure 1). Compared to nanofibers fabricated from raw PLCL, the fiber diameters of blending were less. The average diameter of raw PLCL fibers was 594 ± 119 nm, while that of PLCL/RSSP (75/25), PLCL/RSSP (50/50), PLCL/RSSP (25/75) and PLCL/RSSP (10/90) fibers was 324 ± 100 nm, 222 ± 39 nm, 172 ± 65 nm and 179 ± 41 nm, respectively. This decrease in diameter could be explained by an increase in the conductivity of the blending solution when the two components were blended [51]. RSSP is a typical amphoteric polymer electrolyte, consisting of amino acids containing charged side chains. When RSSP was added to the spinning solution, the charge density of the solution increased, and the stretching effect of the static electricity on the jet was enhanced, resulting in the generation of finer fibers.

### 2.2. Hydrophilicity of the PLCL/RSSP Nanofibrous Membranes

**Figure 2:**
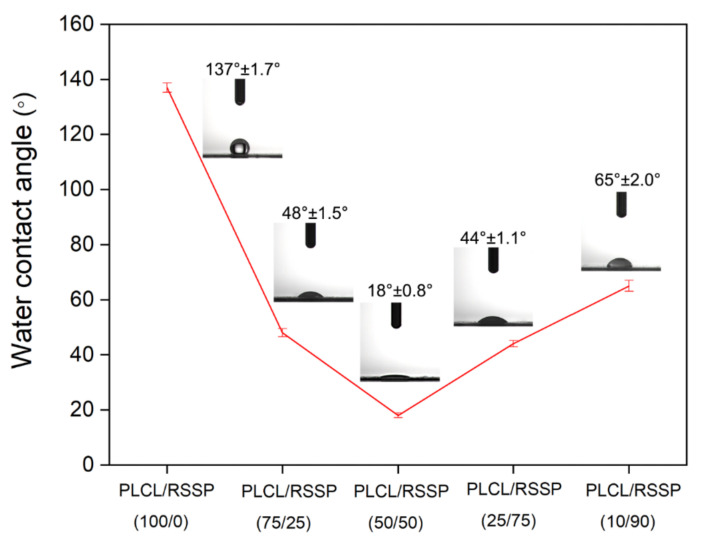
Water contact angles of the nanofibrous membranes.

Hydrophilicity or wettability has been reported to have great influence on the initial adhesion and proliferation of cells on surfaces [53,54]. The dynamic water contact angle was visualized to measure the hydrophilicity of the membrane. The water droplets on the membrane surfaces are shown in Figure 2. The PLCL/RSSP (100/0) membrane showed a hydrophobic surface with the water contact angle of 137 ± 1.7°, which is far more than 90°. This was due to the fact that PLCL molecules contain a large number of hydrophobic groups. When the PLCL was blended with RSSP, the hydrophilicity of the membrane surface increased greatly. This was because the RSSP amino acid sequence is rich in serine, threonine, glutamine and other amino acid residues [52] with side chains containing hydrophilic hydroxyl groups. With the increasing proportion of RSSP, the membranes became more hydrophilic with dramatically decreased contact angles, i.e., the contact angle with PLCL/RSSP (75/25) membrane was 48 ± 1.5° and PLCL/RSSP (50/50) 18.0 ± 0.8°. When the proportion of RSSP was over 50%, the water contact angle increased. This might be due to the different interactions between the two molecular groups when the ratio of two components changes. In previous studies, some nanofibrous membranes electro-spun from blended PLCL and recombinant spider protein showed enhanced hydrophilicity and some did not [51]. In this study, the blending of RSSP significantly increased the hydrophilicity and this would favor cell adhesion and proliferation.

### 2.3. Structure of the PLCL/RSSP Nanofibrous Membranes

**Figure 3:**
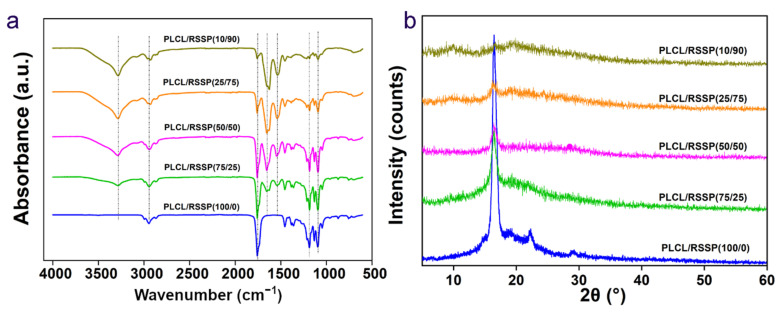
Chemical structure of nanofibrous membrane. (**a**) FTIR spectra. (**b**) WAXD patterns.

The FTIR spectra (4000–600 cm^−1^) of nanofibrous membranes are shown in the Figure 3a. The spectrum of the PLCL/RSSP (100/0) membrane showed the characteristic absorption peaks of raw PLCL. The molecular structure of PLCL contains C = O, C-O, -CH_2_ and -CH_3_. Three representative absorption peaks of PLCL/RSSP (100/0) at 1760 cm^−1^, 1183 cm^−1^ and 1092 cm^−1^ correspond to -COOR and C-O stretching vibrations [55], respectively. The peaks at 2800–3000 cm^−1^ represent the stretching vibration of -CH_2_ and -CH_3_. With the decreasing proportion of PLCL, the absorption peaks at 1092 cm^−1^, 1183 cm^−1^ and 1760 cm^−1^ gradually decreased. On the curve of the membrane of PLCL/RSSP (10/90), the strong peak at 1640 cm^−1^ (amide I band) represented the stretching vibration of the amide bond, the peak at 1550 cm^−1^ (amide II band) represented the presence of N-H and the broad peak at 3300 cm^−1^ was caused by the stretching vibration of N-H and hydrogen-bond interaction. These main peaks indicated the amide I and II bands [56] which represented important functional groups of protein. The absorption of the peaks at 1640 cm^−1^, 1550 cm^−1^ and 3300 cm^−1^ decreased gradually with the decreasing proportion of RSSP.

WAXD patterns of the five samples are depicted in Figure 3b. The spectrum of the raw PLCL/RSSP (100/0) membrane showed Bragg reflections at 16.4° and 22.3°. The well-defined peak centered at 16.4° indicated that the electro-spun PLCL/RSSP (100/0) fibers were mainly crystalline in structure. These diffraction peaks gradually became weaker when the blending proportions of RSSP increased, indicating that PLCL had been converted to the amorphous physical form. With the increasing proportion of RSSP, a few weak broader diffraction peaks appeared, due to Silk II and silk I nanocrystal structures of RSSP. For silk fibers, the microstructure is the nanocrystal embedded in an amorphous matrix [57,58,59,60]. The results suggested that main structures of the blended membranes were more amorphous with higher proportions of RSSP than that in raw PLCL. These difference in microstructure would affect the mechanical properties of the membranes.

### 2.4. Thermal Decomposition Properties of the PLCL/RSSP Nanofibrous Membranes

**Figure 4:**
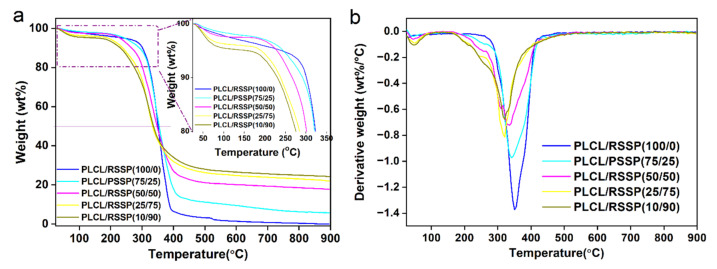
TGA of the PLCL/RSSP nanofibrous membranes. (**a**) TG curves. (**b**) DTG curves.

Thermogravimetric analysis is an important way to study the thermal stability of polymers. Figure 4 shows the TG and DTG curves of PLCL/RSSP nanofibrous membranes. A slight decrease in weights in all curves below 100 °C was due to the vaporization of water inside the nanofibrous membranes. The temperature of the thermal degradation onset point decreased with an increasing proportion of RSSP. A rapid weight loss happened between 250 °C and 350 °C, caused by the thermal decomposition of the material. Meanwhile, when the weight dropped to 50%, the thermal temperature was in the range of 340–380 °C, showing a slight decrease with the increasing proportion of RSSP. All these effects might be due to the side chains of RSSP and its more amorphous microstructure causing a decrease in the temperature of thermal decomposition. Unlike the raw PLCL membrane which was decomposed totally at 900 °C, the blended ones still retained some proportion of weight; with an increasing proportion of RSSP, the residues increased from 5% to more than 20%. The residues of these blended membranes were probably pseudographitic pyroproteins because the beta-sheet structure of silk protein was transformed into carbon hexagonal structure to form pseudographitic crystalline form simply by heating to 350 °C [61]. According to previous reports, recombinant spider silk proteins dissolved in HFIP in spinning dope were mostly in alpha-helix form [52,62]. The beta-sheet structure was transformed from alpha-helix during the electro-spinning process.

Figure 4b shows DTG curves of nanofibrous membranes, revealing the decomposition temperature where the maximum weight loss rate is reached. From the curves, it can be observed that the peak decomposition temperature of raw PLCL nanofibrous membrane reached is at about 350 °C, and that of blended ones decreases slightly with the increasing proportion of RSSP.

### 2.5. Mechanical Properties of the PLCL/RSSP Nanofibrous Membranes

The mechanical properties of the RSSP/PLCL nanofibrous membranes are listed in Table 1 and the representative stress-strain curves are shown in Supplementary Figure S2. The membrane spun from raw PLCL was easy to deform, weak in strength and elastic with great extensibility. The mechanical properties of blended membranes changed greatly. The blending of RSSP decreased the strain at the break significantly as described in a previous report [51]; here, it dropped from 287% to 18% showing a decreasing trend with increasing proportions of RSSP. Compared with the PLCL/RSSP (100/0) membrane, the tensile strength of PLCL/RSSP (75/25) and PLCL/RSSP (25/75) membranes increased greatly to 16.6 MPa and 15.4 MPa, respectively, which was mainly due to the formation of RSSP’s beta-sheet structure during the electro-spinning process [53,63,64]. But the tensile strength of PLCL/RSSP (50/50) membrane showed no difference and that of PLCL/RSSP (10/90) decreased. The secant modulus at 5% showed that the blended membranes were much stiffer than raw PLCL membrane. The variation among the nanofibrous membranes was caused by the different interactions between the molecules causing different microstructures. A recent report showed that RSSP could be spun into fiber with high extensibility of 60–220% with a wet-spinning process [52], but here the blending decreased the extensibility greatly. The reason was that the electro-spinning process lacks stretching in the coagulator or post-stretching which is important for synthetic spider fibers to have improved mechanical properties [52].

The mechanical properties of human skin vary greatly according to age [63], gender, health condition, etc. The modulus covers the range 0.2–18 MPa [63,64]. The tensile strength value ranges from 5 to 30 MPa, and the average strain of skin varies from about 35% to 115% [1]. In other tissue engineering, e.g., vascular tissue engineering, with scaffolds implanted into the patient, mechanical property is important [65] and the scaffold should be comparable to the mechanical performance of the tissues [66]. In skin regeneration, the epithelial sheets or three-dimensional tissue can be cultured in vitro before being grafted on the patient [8]. Among the five membranes, PLCL/RSSP (75/25) membrane possessed the best mechanical properties, comparable to human skin. The tensile strength of PLCL/RSSP (75/25) membranes was 16.6 ± 0.9 MPa and the strain was 81.9 ± 2.2%, which is more similar to human skin than the composite PLCL membranes in previous reports [67,68]. It is thus suggested that the PLCL/RSSP (75/25) nanofibrous membrane would be suitable for skin regeneration in terms of mechanical properties. The remaining assays were performed on the PLCL/RSSP (75/25) membrane with PLCL/RSSP (100/0) membrane as a control.

### 2.6. Hemocompatibility of the PLCL/RSSP Nanofibrous Membranes

**Figure 5:**
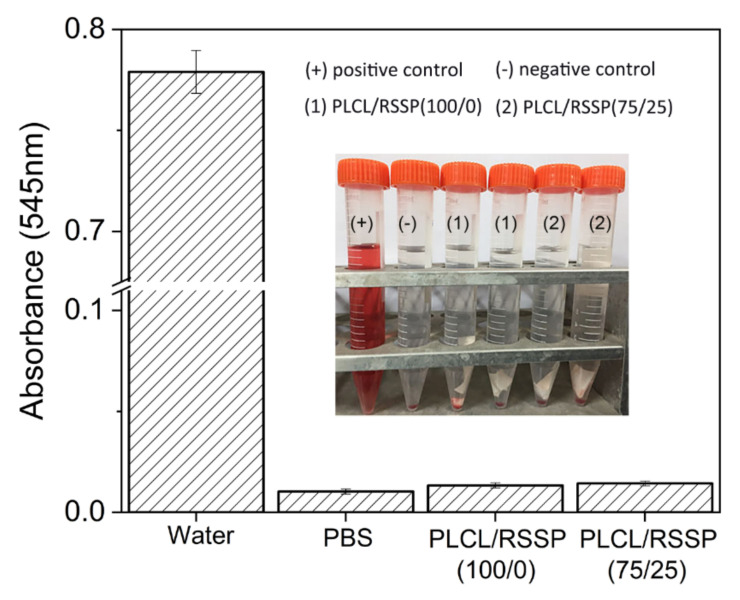
Hemolysis assays of PLCL/RSSP membranes in contact with blood in vitro (*n* = 3). Picture inset shows the hemolytic condition after centrifugation.

The physical and chemical agents on the surface of engineering scaffolds could damage erythrocytes, thereby leading to the release of hemoglobin [69]. The degree of erythrolysis of PLCL/RSSP (75/25) membrane was evaluated and the results of hemolysis assays are shown in Figure 5. The relative hemolysis rates (HR) of the PLCL/RSSP (100/0) and PLCL/RSSP (25/75) membranes were 0.4% and 0.5%, respectively. They were far less than 5%, which is considered safe according to ISO 10993-4 [70]. Previous research has proved that PLCL is nontoxic, tissue-compatible and biocompatible [13,14,15,16] and is a benign material for tissue-engineering scaffolds [13,17,18,19]. Here the results indicated the blended nanofibrous membranes were benign enough to be used as biomaterials for tissue engineering.

### 2.7. Cytocompatibility of the PLCL/RSSP Nanofibrous Membranes

**Figure 6:**
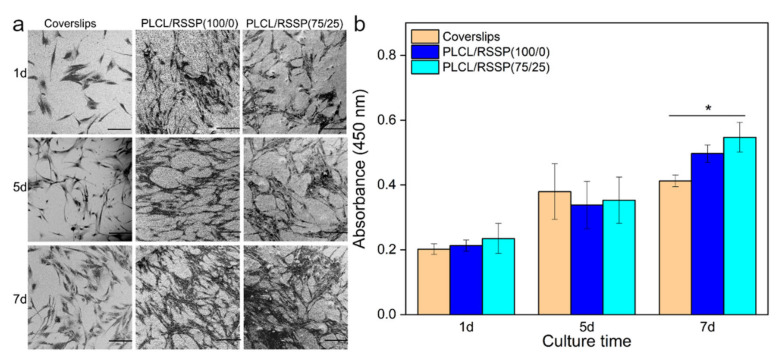
SEM images (**a**) and quantitative analysis (**b**) of the proliferation of hFFCs on the cover slips of PLCL/RSSP (100/0) and PLCL/RSSP (75/25) membranes. (Scale bar: 200 μm. * *p* < 0.05).

The adhesion and proliferation of human foreskin fibroblast cells (hFFCs) seeded on the membranes in vitro were investigated for the cytocompatibility of the nanofibrous membranes. The SEM images (Figure 6a) showed the cell morphologies on the coverslips and the PLCL/RSSP (100/0) and PLCL/RSSP (75/25) membranes after 1, 5 and 7 days’ culturing. The cells on the PLCL/RSSP (100/0) membrane showed poor adhesion compared to other samples. This may be due to the hydrophobic surface of PLCL membrane. Many studies have demonstrated that moderately hydrophilic surfaces promoted the highest levels of cell attachment and growth [51,53]. The morphology of the cells on the PLCL/RSSP (75/25) membrane were spindle-shaped and spread well with obvious pseudopodia, exhibiting similar adhesive morphology to the control cells cultured on the coverslips. This might be due to greatly enhanced hydrophilicity of the PLCL/RSSP (75/25) membrane.

CCK-8 assays were used to evaluate the viability of hFFCs on the nanofibrous membranes. The results on day 1, 5 and 7 after culturing on the nanofibrous membranes are shown in Figure 6b. There was no significant statistical difference between the cells cultured on cover slips and the nanofibrous membranes after five days’ culture. After 7 days, comparing with the ones on the coverslips, the cells proliferated better on the nanofibrous membranes (*p* < 0.05). This was because the nanofibrous membranes offered topographic guidance [26] and their spatial structures have effects on cell proliferation [27] in the long run. Although the cells on the PLCL/RSSP (75/25) membrane proliferated better than on the raw PLCL membrane, there is no significant difference. The results indicate that the PLCL/RSSP (75/25) membrane can support the growth of hFFCs in vitro and has the potential for use as a skin regenerating scaffold. 

### 2.8. Cell Proliferation of the PLCL/RSSP Nanofibrous Membranes with hEGF

Spider silk films provide a promising protein drug delivery matrix [71]. hEGF can stimulate fibroblast cell growth and proliferation. The presence of hEGF in nanofibrous membranes should greatly improve their skin regeneration properties.

**Figure 7:**
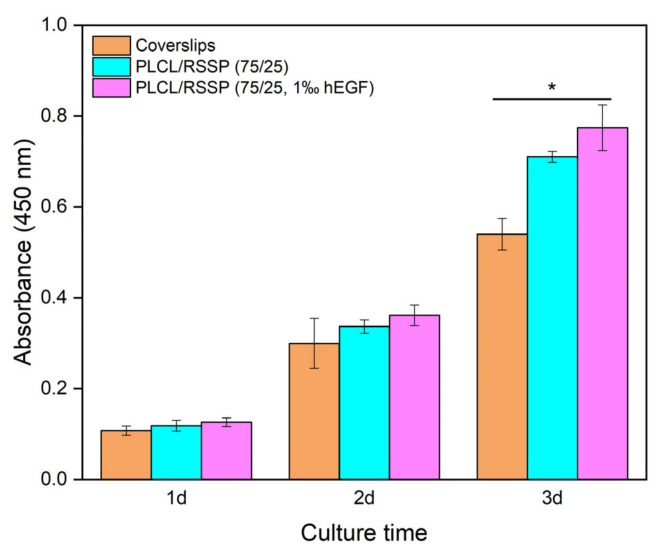
The proliferation of NIH-3T3 cells on the cover slips, PLCL/RSSP (75/25) and PLCL/RSSP (75/25, 1‰ hEGF) membranes. (* *p* < 0.05).

The proliferation of mouse embryonic fibroblast cells (NIH-3T3) was carried out to investigate the hEGF delivery capacity of the PLCL/RSSP nanofibrous membranes. The results of CCK-8 assays are shown in Figure 7. The cells on PLCL/RSSP (75/25, 1‰ hEGF) grew a little bit faster (*p* < 0.05) after day 1 comparing to cells on coverslips, while the cells on PLCL/RSSP (75/25) showed a difference (*p* < 0.05) after day 3, compared to the cells on coverslips. The cells on PLCL/RSSP (75/25, 1‰ hEGF) also grew better (*p* < 0.05) than those on PLCL/RSSP (75/25) after day 2. The results showed that incorporating hEGF into the nanofibrous membranes promoted the proliferation of NIH-3T3 cells, which will improve the performance of the nanofibrous membranes as skin regenerating scaffolds.

### 2.9. Degradability of the PLCL/RSSP Nanofibrous Membranes In Vitro

The biodegradability of the materials affects cell growth, tissue regeneration and host response [14], which is important for most tissue engineering scaffolds [69,72]. The regeneration period of skin is 2 to 3 weeks [1]. The degradation of the nanofibrous membranes was analyzed in vitro for four weeks.

**Figure 8:**
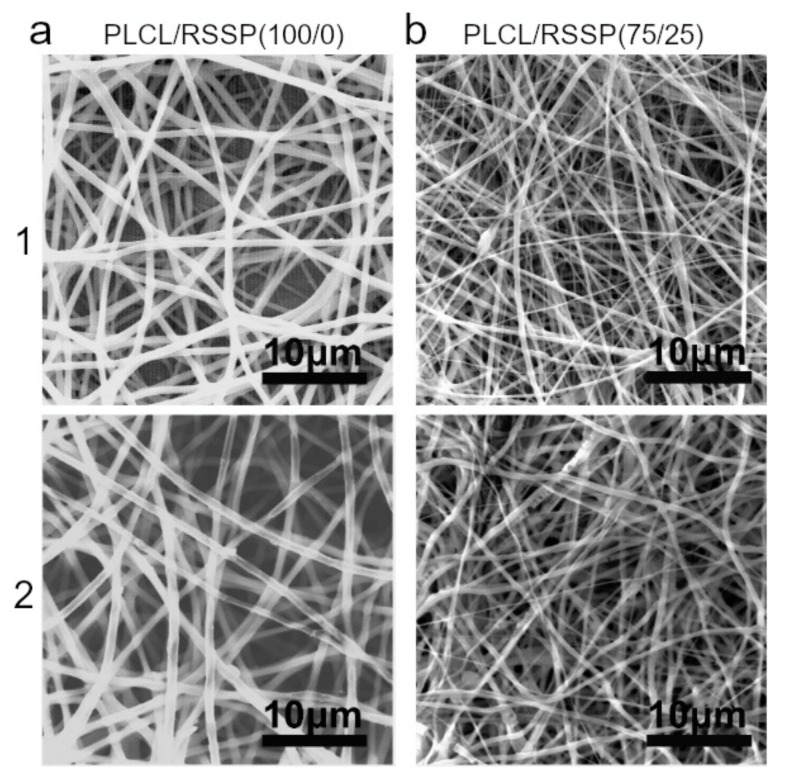
SEM images of degraded membranes. (**a1**) PLCL/RSSP (100/0) membranes before degradation; (**a2**) PLCL/RSSP (100/0) membranes after 4-week degradation; (**b1**) PLCL/RSSP (75/25) before degradation; (**b2**) PLCL/RSSP (75/25) membranes after 4-week degradation.

SEM images of PLCL/RSSP (100/0) and PLCL/RSSP (75/25) membranes after 4-week degradation are shown in Figure 8. Both PLCL/RSSP (100/0) and PLCL/RSSP (75/25) nanofibers remained as round threads without obvious breakages. The weight loss of PLCL/RSSP (100/0) and PLCL/RSSP (75/25) membranes was 3.6 ± 0.2% and 3.8 ± 0.3%, respectively. The results indicated that the degradation of the blended membrane was a little bit faster that of raw PLCL one. It is well accepted that PLCL is degraded basically by non-enzymatic random hydrolytic scission of esters linkages [14]. The weight loss was caused by some of the molecules on the surface being dispersed and dissolved in the medium by the hydrolysis cracking of the polymer chains. Raw PLCL membrane was hydrophobic and mainly in crystalline structures; therefore, the rate at which the membrane was permeated by the buffer and degradation rate were both slow. The PLCL/RSSP (75/25) membrane was hydrophilic with an amorphous structure. Amorphous regions were first attacked by water because they were easier for water to penetrate [14].

## 3. Materials and Methods

### 3.1. Materials

Poly(l-lactic acid-co-ε-caprolactone) (PLCL, molar ratio of PLLA: PCL = 50:50) was purchased from Jinan Daigang Biomaterial Co., Ltd. (Jinan, China). 1, 1, 1, 3, 3, 3-hexafluoro-2-propanol (HFIP) was acquired from Shanghai Darui Finechemical Co., Ltd. (Shanghai, China). Anhydrous dimethyl sulfoxide (DMSO) was purchased from Changshu Hongsheng Chemical Reagent Co., Ltd. (Changshu, China). Paraformaldehyde (POM), human epidermal growth factor (hEGF) and Cell Counting Kit -8 (CCK-8) were purchased from Biyotime Institute of Biotechnology (Shanghai, China). Dulbecco’s Modified Eagle’s Medium (DMEM), fetal bovine serum (FBS), phosphate buffer saline (PBS) from Mesgen Company (Shanghai, China). Penicillin-streptomycin and trypsin were purchased from Shanghai Yuanxiang Medical Equipment Co. Ltd. (Shanghai, China). All chemicals were used without further purification. Deionized water used in all experiments was purified using a Milli-Q Plus 185 water purification system (Millipore, Burlington, MA, USA) with resistivity higher than 18 MΩ cm. Human foreskin fibroblast cells (hFFCs) and mouse embryonic fibroblast cells (NIH-3T3) were obtained from Shanghai Institute of Biochemistry and Cell Biology (SIBCB, CAS, China).

### 3.2. Preparation of Nanofibrous Membranes

The recombinant spider silk protein (RSSP, MW: 93.4 kDa) was produced as described in a previous work [52]. Briefly, *Escherichia coli* Strain BL21 (DE3) cells transformed with expressing plasmids were cultured in Luria broth medium containing 50 µg∙mL^−1^ ampicillin in a shaker at 37 °C and 200 rpm. When the optical density OD_600_ reached 0.6–0.8, the cells were induced with isopropyl β-D-1-thiogalactopyranoside (IPTG) and then incubated at 16 °C and 180 rpm for 16 h. The cells were harvested by centrifugation (4 °C, 4000× *g*, 20 min), suspended in lysis buffer (20 mM Tris-HCl, 100 mM NaCl, pH 8.0) and then lysed. After removing the supernatant by centrifugation (4000× *g*, 40 min, 4 °C), the proteins of interest which were present in the insoluble fraction were re-suspended in washing buffer (4M urea, 20 mM Tris-HCl, pH 8.0, 100 mM NaCl) and sonicated for 30 min in an ice bath. Then the sample was centrifuged (4000× *g*, 40 min, 4 °C) and the pellets were solubilized in dissolution buffer (8 M urea, 20 mM Tris-HCl, pH 8.0, 100 mM NaCl) followed by a centrifugation (12,000× *g*, 25 °C, 30 min) removing any insoluble material. The protein solution was gradient-dialyzed against dialysis buffer and finally against de-ionized water. The final solution was freeze-dried.

The nanofibrous membranes were prepared by an electro-spinning process (Supplementary Figure S1) similar to previous reports [69,73]. Briefly, PLCL and lyophilized RSSP powder were dissolved in HFIP with vigorous magnetic stirring for 24 h to harvest pellucid solution as the spinning dope. The blending ratios of PLCL/RSSP were 100/0, 75/25, 50/50, 25/75 and 10/90 (*w*/*w*). The spinning dope with the total concentration of 6% (*wt*/*v*) polymer was ejected with an LSP01-3A syringe pump (Longer Precision Pump Co. Ltd., Shanghai, China) through an 18 G syringe needle at the speed of 0.6 mL∙h^−1^. The applied high voltage was set at 12 kV. A paperboard wrapped with aluminum foil was used as a collector and positioned horizontally and grounded perpendicularly 14 cm from the needle tip. After electrospinning, collected nanofibrous scaffolds were crosslinked by ethanol vapor for 24 h and then dried in a vacuum desiccator for 24 h before further research. All the electro-spinning processes were carried out at around 25 °C and 50 ± 2% relative humidity.

The nanofibrous membranes containing hEGF were prepared similarly to the above. hEGF was added to HFIP, dissolving together with PLCL and RSSP for 24 h. The final concentration of hEGF in the spinning dope was 1‰ (*wt*/*v*). The same procedures were followed to prepare membranes for further experiments.

### 3.3. Scanning Electron Microscopy (SEM)

The morphology of the nanofibrous membranes was investigated with a scanning electron microscope (SEM) (Phenom XL, Netherland) at an acceleration voltage of 10 kV after being sputter-coated with gold for 45 s. The distribution of fiber diameters was analyzed by ImageJ (National Institutes of Health, Bethesda, MD, USA) from 100 nanofibers on SEM images randomly selected for each sample at magnification of 5000.

### 3.4. Water Contact Angle Measurement

Hydrophilicity of the nanofibrous membranes was determined by water contact angles measured with a drop shape analyzer DSA30 (KRUSS, Hamburg, Germany) with the sessile drop technique water contact angle. All data were analyzed with ADVANCE-drop shape software to determine the contact angles. The final data were averaged from at least three measurements from three samples of each membrane.

### 3.5. Fourier Transform Infrared (FTIR) Spectroscopy

The chemical bonds and functional groups present in the nanofibrous membranes were analyzed from the spectra recorded with a Nicolet 6700 FITR spectrometer (Thermo Fisher, Waltham, MA, USA) in the range of 4000–600 cm^−1^ in attenuated total reflection mode at room temperature. For each spectrum, 64 scans were averaged with a resolution of 1 cm^−1^.

### 3.6. Thermogravimetric Analysis (TGA)

Thermal properties were measured by a thermo-gravimetric instrument TG209F 1 (TA Instruments Corp., New Castle, DE, USA). Samples (3–5 mg) were heated from 29 to 900 °C at a heating rate of 10 °C min^−1^ under a flow of nitrogen (40 mL∙min^−1^).

### 3.7. Wide-Angle X-ray Diffraction (WAXD)

Wide-angle X-ray diffraction (WAXD) patterns of nanofibrous membranes were collected with a D/max-2550VB+/PC X-Ray Diffractometer (Rigaku international Corp., Tokyo, Japan) Cu/Kα radiation (40 kV /150 mA, λ _CuKα_ = 1.54056 Å) over the 2θ range of 5° to 60°.

### 3.8. Mechanical Testing

The mechanical properties of nanofibrous membranes were tested by a universal material tester H5K-S (Hounsfield, UK) with a crosshead speed of 30 mm∙min^−1^ with gauge length of 30 mm. All the nanofibrous membranes were cut into strips (50 mm × 10 mm); four strips from different sites of each fibrous membrane were chosen for testing and the results averaged from four tests. The stress and strain data were calculated according to previous literature [74,75].

### 3.9. Hemolysis Assays

Before the assays, the membranes (3 cm × 1 cm) were soaked in 75% ethanol for half an hour for sterilization, then rinsed three times with sterile water. Healthy red blood cells (HRBCs) were obtained from fresh New Zealand white rabbit blood containing 3.8% (*w*/*v*) sodium citrate as anticoagulant (the ratio of anticoagulant to blood is 1:9, *v*/*v*). The assays were performed according to the previous literature [76,77]. In brief, the HRBCs were obtained by centrifuging the fresh blood (1200× *g* for 10 min). The precipitates were washed with PBS for 5 times to completely remove the serum. The HRBCs were diluted 10 times with PBS before hemolysis assay. The diluted HRBCs (0.2 mL) mixed with PBS solution (10 mL) with a total volume of 10.2 mL were added into a centrifugal tube with one strip of nanofibrous membrane. The diluted HRBCs (0.2 mL) were also mixed with 10 mL water as a positive control and 10 mL PBS buffer as a negative control for comparison, respectively. After a gentle shaking, all samples were incubated at 37 °C for 2 h and were gently shaken every half an hour. All the HRBCs suspensions were taken away carefully and were centrifuged at 2000× *g* for 5 min. The absorbance at 545 nm of the supernatant (hemoglobin) was determined by Lambda 25 UV–Vis spectrophotometer (Perkin Elmer, Waltham, MA, USA). The hemolysis rate (HR) was defined as the following formula: HR = (SA−NA)/(PA−NA) ×100%, where SA, PA, and NA stand for the supernatant absorbency of the experimental sample, the positive control and the negative control, respectively. Mean and standard deviations of the triplicate centrifugal tubes for each sample were calculated.

### 3.10. Cell Attachment and Proliferation on the Nanofibrous Membranes

Membrane samples were cut into discs of 14 mm in diameter and placed into 24-well plates individually and secured by stainless steel rings. Before cell seeding, the nanofibrous membranes were sterilized by exposure to 400 mgL^−1^ ethylene oxide for 12 h. The fibroblast cells (hFFCs or NIH-3T3) were seeded with a density of 1.0 × 10^4^ cells per well and cultured in Dulbecco’s modified Eagle’s medium (DMEM) with 10% fetal bovine serum and 1% antibiotic-antimycotic in an atmosphere of 5% CO_2_ and 37 °C and the medium was replenished every two days.

Cell Counting Kit-8 (CCK-8) was employed to evaluate the viability of the proliferated hFFCs cultured on nanofibrous membranes, three samples for each group. The time points for testing were set after 1, 5, and 7 days (hFFCs) or 1, 2, and 3 days (NIH-3T3), respectively. At each time point, the culture medium was removed, and the samples were rinsed with PBS and fresh medium was added into the wells. Then CCK-8 assays were operated according to the manufacturer’s protocol. The measurements were performed in triplicate.

Additionally, for examining the spread and attachment of hFFCs cultured on the nanofibrous membranes, seeded membranes were rinsed three times with PBS and then fixed with 4% paraformaldehyde overnight. Fixed samples were dehydrated by gradient ethanol (30%, 50%, 70%, 80%, 90%, 95% and 100%). After being dried under vacuum, the membranes were coated with gold for SEM observation.

### 3.11. Degradation of the Nanofibrous Membranes

The hydrolytic degradation of nanofibrous membranes was investigated in vitro over the period of 4 weeks. Dried nanofibrous membranes were cut into slices and the initial weight (W_1_) was recorded. Then they were soaked in 10 mL PBS (10 mM, pH 7.0) containing 0.002% (*w*/*v*) sodium azide. Samples were sealed up and incubated in a continuous shaker with 120 rpm at 37 °C. The buffer was changed every week. After four weeks, the membranes were taken out from PBS, rinsed with distilled water and then processed with vacuum freeze drying for weighing (W_2_). The mass loss ratio was calculated as follows: mass loss ratio (%) = (W_1_−W_2_)/W_1_ × 100% [51]. The membranes were also coated with gold for SEM observation.

### 3.12. Statistical Analysis

In all the experiments, all results are represented as the mean ± standard deviation [78]. The data was analyzed by one-way ANOVA, followed by Tukey’s test for the evaluation of specific differences with Origin 8.0 (Origin lab Inc., Northampton, MA, USA). A value of 0.05 was selected as the significance level, and the data was indicated with (*) for *p* < 0.05.

## 4. Conclusions

In this paper, we used blended poly(l-lactide-co-ε-caprolactone) (PLCL) and recombinant spider silk protein (RSSP) in different ratios to prepare potential skin regeneration scaffold material with an electro-spinning process. The blending of RSSP with PLCL changed various properties of the nanofiber membranes, making the composite PLCL/RSSP membranes more suitable to be skin regeneration scaffolds than other PLCL composite membranes. Among the blended membranes, the PLCL/RSSP (75/25) membrane possessed good qualities for use as the scaffold for skin cell culturing in vitro. The diameter of nanofibers in the PLCL/RSSP (75/25) membrane decreased to 324 ± 100 nm comparing with raw PLCL nanofibers (594 ± 119 nm). The membrane became hydrophilic with the presence of RSSP and the dynamic water contact angle was 48 ± 1.5°, whereas the raw PLCL membrane was hydrophobic with a water contact angle of 137 ± 1.7°. The addition of RSSP also changed the mechanical properties of the membranes. The tensile strength of PLCL/RSSP (75/25) membranes was 16.6 ± 0.9 MPa and the strain was 81.9 ± 2.2%. The PLCL/RSSP (75/25) membranes showed very similar mechanical properties to human skin, which was much better than the composite PLCL membranes reported in other works [67,68]. The hemolysis assays and cytocompatibility assays showed higher biocompatibility of the PLCL/RSSP (75/25) membrane than the raw PLCL one. The loading of a small peptide (hEGF) promoted cell proliferation on the membrane. Since RSSP can be treated with chemical and genetical modification [44], better scaffolds could be prepared in future based on such membranes for application to skin-tissue engineering.

## Figures and Tables

**Table 1:** Mechanical properties of nanofibrous membranes (4 times for each membrane).

	Stress (MPa)	Strain (%)	Secant Modulus at 5% (MPa)
PLCL/RSSP (100/0)	7.6 ± 0.3	287.7 ± 26.3	6.7 ± 1.0
PLCL/RSSP (75/25)	16.6 ± 0.9	81.9 ± 2.2	45.6 ± 0.8
PLCL/RSSP (50/50)	8.0 ± 0.8	46.2 ± 5.2	64.2 ± 5.3
PLCL/RSSP (25/75)	15.4 ± 0.3	36.1 ± 4.4	205.1 ± 22.9
PLCL/RSSP (10/90)	6.5 ± 0.3	18.0 ± 7.40	111.8 ± 9.3

## Data Availability

Not applicable.

## Supplementary Materials

The following supporting information can be downloaded at: https://www.mdpi.com/article/10.3390/ijms232214055/s1.

## Abbreviations

RSSP	recombinant spider silk protein
PLCL	poly(l-lactide-co-ε-caprolactone)
SEM	scanning electron microscopy
FTIR	Fourier transform infrared spectroscopy
WAXD	wide angle X-ray diffraction
TGA	thermo-gravimetric analysis
hFFCs	human foreskin fibroblast cells
NIH-3T3	mouse embryonic fibroblast cells (NIH-3T3)
hEGF	human epidermal growth factor
DTG	derivative thermogravimetry
TG	thermogravimetry
HR	hemolysis rates
