# Supplementary material for: Preparation and Characterization of Nanofibrous Membranes Electro-Spun from Blended Poly(l-lactide-co-ε-caprolactone) and Recombinant Spider Silk Protein as Potential Skin Regeneration Scaffold
Source: Int J Mol Sci. 2022 Nov 14;23(22):14055. doi: 10.3390/ijms232214055 (PMC9698895; doi:10.3390/ijms232214055)
Supplement: Supplementary file 1 [file ijms-23-14055-s001.zip › ijms-2001880-supplementary.pdf]

# Supporting Information

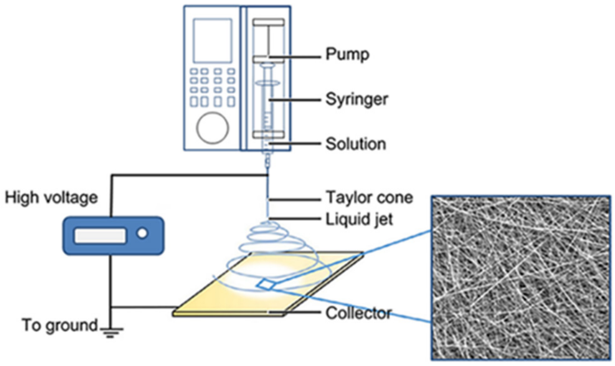


**Figure S1** Schematic illustration of electro-spinning process.

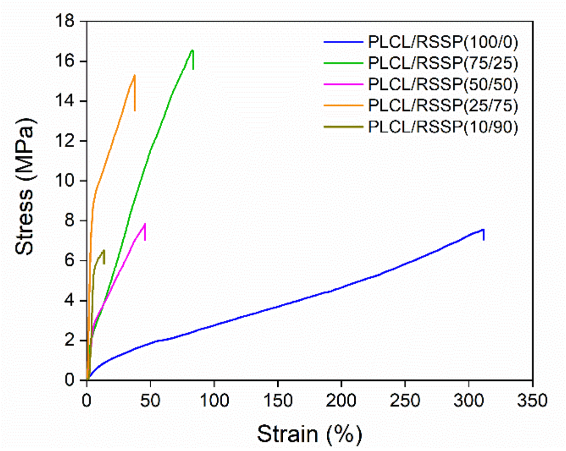


**Figure S2** Representative stress-strain curves of PLCL/RSSP nanofibrous membranes
